# The risk of acute coronary syndrome in rheumatoid arthritis in relation to tumour necrosis factor inhibitors and the risk in the general population: a national cohort study

**DOI:** 10.1186/ar4584

**Published:** 2014-06-18

**Authors:** Lotta Ljung, Johan Askling, Solbritt Rantapää-Dahlqvist, Lennart Jacobsson

**Affiliations:** 1 Rheumatology, Division of Medicine, Department of Public Health and Clinical Medicine, University Hospital, SE-901 87 Umeå, Sweden; 2 Department of Medicine, Clinical Epidemiology Unit, Karolinska Institute, SE-171 77 Stockholm, Sweden; 3 Department of Rheumatology and Inflammation Research, Institute of Medicine, Sahlgrenska Academy at University of Gothenburg, Guldhedsgatan 10, SE-405 30 Gothenburg, Sweden

## Abstract

**Introduction:**

The elevated risk of ischaemic heart disease in patients with rheumatoid arthritis (RA) has been linked to inflammation and disease severity. Treatment with tumour necrosis factor inhibitors (TNFis) is often effective in reducing disease activity and could possibly modify cardiovascular risk. Our objective in the study was to evaluate the risk of acute coronary syndrome (ACS) in patients with RA treated with TNFis compared with the risk among biologic-naïve RA patients and the general population.

**Methods:**

By linkage of the Swedish National Patient Register and the Swedish Biologics Register, we identified a cohort of patients who were started on their first biologic, a TNFi, between 2001 and 2010 (*N* = 7,704), and a cohort comprising matched biologic-naïve RA patient referents at a 3:1 ratio. Furthermore, a matched comparator cohort (5:1 ratio) was extracted from the Swedish population register. The incidence rates of a first ACS event were calculated and compared between cohorts using Cox proportional hazards regression in three different risk windows: ‘ever-exposed’, ‘actively on TNFi’ and ‘short-term exposure’ (active treatment maximized to 2 years). The models were adjusted for disease duration, joint surgery, comorbidity and socioeconomic factors, and, in a sensitivity analysis including a subpopulation started on therapy beginning 1 January 2006 or later, for dispensed drugs.

**Results:**

Based on 221 events in 7,704 patients (comprising 32,621 person-years) treated with TNFi biologics, the hazard ratio ((HR); ever-exposed) for ACS among the TNFi-exposed RA patients compared with biologic-naïve RA patients was 0.8 (95% confidence interval (CI) = 0.7 to 0.95). In comparison with the general population referents, statistical analysis using fully adjusted models resulted in a HR of 2.0 (95% CI = 1.8 to 2.3) for biologic-naïve RA patients and a HR of 1.6 (95% CI = 1.4 to 1.9) for the TNFi-exposed group. Similar risk estimates were obtained using the other two risk windows. A sensitivity analysis in which we compared the TNFi-exposed patients included from 1 January 2006 onward with biologic-naïve patients resulted in a HR (ever-exposed) of 0.7 (95% CI = 0.5 to 1.0).

**Conclusions:**

RA patients treated with TNFi had a lower risk of ACS compared with biologic-naïve RA patients. Compared with the general population, the risk among patients with RA was elevated, although the difference was less pronounced among the TNFi-exposed patients. This finding could be attributable to the TNFi as such, or it could correspond to a lower degree of inflammation in the TNFi-treated group.

## Introduction

Rheumatoid arthritis (RA) is associated with an increased risk for cardiovascular disease (CVD) [1]. The level of increased risk, approximately a doubled risk for myocardial infarction compared with the general population, is in parity with the risk in patients with diabetes mellitus [2-6]. The relative contributions of traditional risk factors to the CVD risk in RA is lower than that in the general population, indicating the importance of factors related to the RA disease itself or to its treatment [7-10]. Control of the systemic inflammation associated with RA, together with identification and management of traditional risk factors, might therefore prove to be an effective measure to prevent myocardial infarctions in patients with RA [11].

Investigators in several published observational studies have examined the possible effect of disease-modifying antirheumatic drugs (DMARDs) and/or biologic therapies (mainly tumour necrosis factor inhibitors (TNFis)) on the risk for ischaemic coronary events in RA [12-21]. A statistically significant reduction in the risk for ischaemic heart disease was observed by researchers in three studies [12,14,16]. In the other studies [13,15,17-21], no associations between biologic therapy and the risk for ischaemic heart disease were observed, leaving unanswered the important question whether TNFis may indeed improve not just RA disease activity, but ultimately also the cardiovascular comorbidity profile in RA.

In a previous study on the risk for acute coronary syndrome (ACS) after TNFi exposure in patients with early RA (average RA disease duration of 1.4 years at the start of TNFi therapy), we observed a 20% lower risk for ACS among patients started on TNFi therapy compared to patients treated otherwise (relative risk (hazard ratio (HR)) = 0.80, 95% confidence interval (CI) = 0.52 to 1.24) [15]. The lack of a significantly lower risk among the TNFi-exposed patients might be due to lack of a true protective effect or a lack of precision of the data, but it also might be due to the difference in the average level of accrued disease control (whether obtained through TNFi or alternative therapies) being too small to allow for a ‘protective’ effect of TNFi to be observed. Our purpose in the present study was therefore to evaluate the role of TNFi treatment on the risk for ACS in a large national cohort that mainly included patients with established RA.

## Methods

### Setting

For this study, in which we employed a matched, prospective, population-based cohort design, the study populations were identified from among the individuals in the Swedish Biologics Register, the Swedish National Patient Register and the Swedish population register (Statistics Sweden). The Swedish setting, including its population-based registers, have previously been described elsewhere [22-24].

In brief, Statistics Sweden holds data on immigration, emigration, vital status and residency, as well as data on socioeconomic factors such as civil status and education level, for all residents in the country. The Swedish National Patient Register is a population-based register with information on inpatient care since 1964 and full coverage since 1987. In addition, since 2001, outpatient visits to both public and private caregivers other than in a primary care setting have also been included in the register. The register is updated annually. For this study, data through 31 December 2010 were available. The information in the National Patient Register is divided into four groups: (1) patient data such as the unique individual personal identification number and place of residence, (2) geographical data such as hospital and department, (3) administrative data such as visit dates or dates of admission and discharge and (4) medical data such as main and secondary diagnoses. The discharge diagnoses are coded by the discharging physician according to the codes assigned by the International Classification of Diseases (ICD), revisions 7 to 10. The Swedish Biologics Register has been in use since 1998 for follow-up of patients with RA and other rheumatologic conditions who are older than 18 years of age and were started on a biologic treatment. All public and private rheumatology clinics and practices report data to be included in the register, with an estimated national coverage of around 90% regarding prescribed TNFi drugs (H Wadström, personal communication) [25]. Furthermore, we used data extracted from the Swedish Cause of Death Register, which contains information on dates and causes of death for all residents who have died since 1961, and the Prescribed Drug Register, which has information on prescribed drugs for ambulatory care dispensed by Swedish pharmacies since July 2005.

### Study population

Patients with two or more clinical visits or hospitalizations for which RA was listed as the main or contributory diagnosis in the National Patient Register, of which at least one was recorded in the Outpatient Register between 2001 and 2010 at an internal medicine or rheumatology department, were defined as having RA. ICD codes are listed in Additional file 1. This RA definition has previously been shown to result in an RA disease prevalence of approximately 0.6% [26]. Data were extracted from the Swedish Biologics Register for patients older than 18 years of age who had RA according to this definition, were started on treatment with a first-ever biologic (a TNFi) between 1 January 2001 and 31 December 2010 and had no diagnosis of ischaemic or congestive heart disease recorded in the National Patient Register prior to treatment start (*N* = 7,704). We hereinafter refer refer to these patients as *index patients*. For each index patient, we identified up to three randomly assessed, matched referents with RA (according to our definition above) who were alive and unexposed to TNFi at the time point for the start of TNFi for the index patient and who did not have a previous diagnosis of ischaemic or congestive heart disease. The matching date we assigned to these matched but biologic-naïve referent patients was the date of start of TNFi treatment of their index patient. The matching was performed in four steps (with each referent being assigned to only one index patient). (1) We used a 1:2 matching algorithm based on sex, age in 5-year strata and county of residence. (2) For any index patient not assigned two referents in step 1, we matched on sex, age in 10-year strata and county of residence. (3) For index patients still without two referents, we matched by sex and by age in 10-year strata. (4) We repeated steps 1 to 3 to find a third referent for each index patient. In total, 23,112 RA referents (the biologic-naïve RA cohort) were identified. From among the individuals in the Swedish population register, we identified five general population referents without any previous diagnosis of ischaemic or congestive heart disease, who were randomly assigned and matched for sex, age in 5-year strata and county of residence to each index patient (*N* = 38,520).

The Regional Ethical Review Board in Stockholm approved the study and waived the requirement for individual consent for this register-based study.

### Follow-up

The follow-up for each individual started at the matching date and ended at death, emigration, 31 December 2010, the time point for first ACS event or (for the biologic-naïve referents) at the reported start date for any biologic agent, whichever came first. Three different risk windows were defined as follows: (1) ‘ever-exposed’, with all follow-up time evaluated regardless of treatment discontinuation; (2) ‘actively on TNFi’, including the follow-up time until the reported stop date for the TNFi plus 90 days and the corresponding date for the referents; and (3) ‘short-term exposure’, defined as actively on a TNFi but with a maximum of 2 years of follow-up. Switching from one TNFi drug to another was considered continuous treatment if the interval between treatments did not exceed 90 days. In accordance with the prospective design of our study, if a previously unexposed biologic-naïve RA patient was started on treatment with a TNFi, he or she was included in the TNFi-exposed cohort from that date.

### Definition of events and covariates

‘Outcome’ was defined as any first-ever ACS event, which in turn was defined as a primary discharge diagnosis of acute myocardial infarction or unstable angina pectoris, or as acute myocardial infarction being the underlying cause of death. For discharge diagnoses, the date of admission to hospital was considered the event date. This outcome definition has previously been validated in a Swedish early RA cohort, with a positive predictive value of 95% [15].

From the National Patient Register, we extracted in- and outpatient data since 1987 and 2001, respectively, on any previous hospitalizations or visits with a main or contributory diagnosis of chronic obstructive pulmonary disease, diabetes mellitus, hypertension, cerebrovascular disease and other atherosclerotic disease, as well as the number of previous hospital admissions during the 10 years antedating the matching date, any joint surgery (knee prosthesis, hip prosthesis, shoulder prosthesis, foot surgery, hand surgery) or hospitalization for infectious disease prior to inclusion.

‘Duration of RA disease’ was defined as more than 10 years if, at the matching date, the time since the first entry of an RA diagnosis in the National Patient Register exceeded 10 years. Data on socioeconomic factors (education level, number of days on sick leave and disability pension in the year before the index date) were retrieved from the Statistics Sweden database.

For a sensitivity analysis including index patients who started TNFi on 1 January 2006 or later and their matched referents, we extracted information on dispensed drugs during the 6 months before the matching date (acetylsalicylic acid, corticosteroids, DMARDs, lipid-lowering drugs, treatment for hypertension or diabetes, nonsteroidal anti-inflammatory drugs (NSAIDs) and coxibs) from the Swedish Prescribed Drug Register.

### Statistical methods

Incidence rates for ACS in each cohort were calculated as the number of observed events divided by the corresponding person-time of follow-up. The relative risks (HRs) for ACS in patients exposed to a first TNFi were estimated by Cox proportional hazards regression, in which we compared patients exposed to a first TNFi with the matched RA referents and with the general population referents, respectively. In the regression models in which we used time since start of follow-up as the time scale, we adjusted for sex, age, county of residence, inclusion year, presence of any diagnoses indicating previous cardiovascular risk factors (diabetes mellitus, hypertension and previous cerebrovascular and/or other atherosclerotic disease), education level, sick leave and disability pension during the year before inclusion, joint surgery, disease duration, previous infections and chronic pulmonary disease. In sensitivity analysis restricted to TNFi start and matched comparators 2006 or later, we further adjusted the regression models for dispensed medications. In these analyses, adjustments for further cardiovascular risk factors such as treatment for hypertension, diabetes and hyperlipidaemia, as well as treatment with corticosteroids, NSAIDs and coxibs, were possible by extracting from the Swedish Prescribed Drug Register (in operation since July 2005) information on dispensed prescribed drugs during the 6-month period immediately antedating study inclusion.

Analyses were performed using SAS 9.3 software (SAS Institute, Cary, NC, USA). Survival analyses with Kaplan-Meier curves were performed using IBM SPSS Statistics for Windows version 20.0 software (IBM, Armonk, NY, USA).

## Results

The clinical and demographic characteristics of the cohorts at the start of follow-up are given in Table 1. The median (and mean) follow-up times in the cohort of TNFi-exposed RA patients were 3.9 (4.2) years for the ‘ever-exposed’ cohort, 2.4 (3.1) years for the ‘actively on TNFi’ cohort and 2.0 (1.5) years for the risk window ‘short-term exposure’ cohort. The corresponding figures for the biologic-naïve cohort were 2.7 (3.3), 2.4 (2.4) and 2.0 (1.5) years, respectively, and for the general population comparator cohort 4.1 (4.3), 2.3 (3.1) and 2.0 (1.7) years, respectively. A majority of the TNFi-exposed patients were started on infliximab (39.5%), etanercept (37.1%) or adalimumab (22.3%) as their first TNFi, and only a small number of patients received one of the more recent drugs as their first TNFi (0.8% for golimumab and 0.4% for certolizumab pegol).

**Table 1:** **Clinical and demographic characteristics of study cohorts at start of follow-up**^**a**^

**Clinical and demographic characteristics**	**RA patients starting TNFi (*N*** **= 7,704)**	**Biologic-naïve RA referents (*N*** **= 23,112)**	**General population referents (*N *** **=38,520)**
Female sex	5,845 (76)	17,535 (76)	29,225 (76)
Age, mean ± SD (yr)	57.1 ± 12.1	57.3 ± 12.2	57.2 ± 12.2
RA duration >10 yr	1,509 (19.6)	3,864 (16.7)	
Comorbidity, previous diagnosis			
Diabetes	379 (4.9)	1,251 (5.4)	1,342 (3.5)
Hypertension	833 (10.8)	2,689 (11.6)	2,571 (6.7)
Chronic obstructive pulmonary disease	196 (2.5)	653 (2.8)	450 (1.2)
Cerebrovascular disease	177 (2.3)	696 (3.0)	938 (2.4)
Other atherosclerotic disease^b^	505 (6.6)	1,803 (7.8)	1,774 (4.6)
Infections (hospitalization)	1,675 (21.7)	5,287 (22.9)	5,487 (14.2)
Number of days in hospital within 10 years before inclusion, median (IQR)	4 (0 to 17)	3 (0 to 14)	0 (0 to 5)
Previous joint surgery^c^	1,974 (25.6)	4,669 (20.2)	1,392 (3.6)
Education level attained			
≤9 years or less	2,197 (28.5)	7,062 (30.6)	10,303 (26.8)
10 to 12 years	3,494 (45.4)	10,473 (45.3)	16,796 (43.6)
>12 years	1,983 (25.7)	5,455 (23.6)	11,182 (29.0)
Any disability pension in year before inclusion^d^	2,601 (33.8)	7,195 (31.1)	4,993 (13.0)
Sick leave the year before inclusion^e^			
During part of year (14 to 349 days)	2,091 (27.1)	5,075 (22.0)	4,319 (11.2)
Whole year (≥350 days)	240 (3.1)	596 (2.6)	386 (1.0)

^a^RA, Rheumatoid arthritis; TNFi, Tumour necrosis factor inhibitor. Values are number of patients (%) unless otherwise stated. ^b^‘Other atherosclerotic disease’ includes arterial occlusion and stenosis of cerebral and precerebral arteries, transient ischaemic attack, cerebral atherosclerosis, late effects of cerebrovascular disease, atherosclerosis, aortic aneurysm, other aneurysm or dissection, peripheral arterial disease, arterial embolism or thrombosis, and atherosclerosis with gangrene. ^c‘^Previous joint surgery’ includes knee prosthesis, hip prosthesis, shoulder prosthesis, foot surgery and hand surgery. ^d‘^Disability pension’ is 25% to 100% of any period of time during the year before inclusion. ^e^The registered number of days on sick leave also includes work-free days for any sick leave longer than 5 days. Periods of sick leave lasting 14 days or less are not included in the Statistics Sweden database.

During follow-up (‘ever-exposed’), a total of 221 ACS events were observed, including 175 (79.2%) primary diagnoses of myocardial infarction, 25 cases (11.3%) of unstable angina and 21 (9.5%) events with acute myocardial infarction recorded as the underlying cause of death.

**Figure 1:**
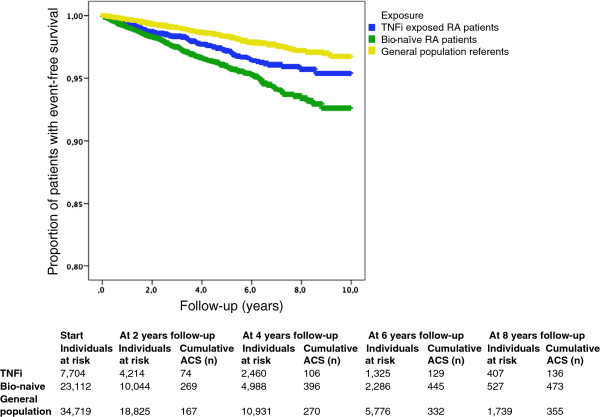
**Event-free survival.** Kaplan-Meier curves illustrating the proportion of individuals with event-free survival among rheumatoid arthritis (RA) patients exposed to tumour necrosis factor inhibitors (TNFi) and matched comparator cohorts of biologic-naïve RA patients and general population referents using the risk window ‘actively on TNFi’. ACS, Acute coronary syndrome.

In all risk windows, the crude incidence rates were lower in the TNFi-exposed cohort compared with the biologic-naïve RA cohort (Table 2). Kaplan-Meier curves displayed a higher event-free survival in patients with RA receiving TNFi treatment compared to the biologic-naïve RA cohort. The event-free survival rate was lower compared to the general population comparator for both TNFi-exposed and biologic-naïve RA patients (Figure 1).

**Table 2:** **Crude incidence rates for acute coronary syndrome in study cohorts**^**a**^

	**TNFi-exposed RA patients (*N*** **= 7,704)**		**Biologic-naïve RA patients (*N*** **= 23,112)**		**General population referents (*N*** **= 38,520)**	
**Risk window**^**b**^	**ACS/person-years**	**Crude IR**	**ACS/person-years**	**Crude IR**	**ACS/person-years**	**Crude IR**
Ever exposed to TNFi	221/32,621	6.8 (5.9 to 7.7)	680/75,268	9.0 (8.4 to 9.7)	602/165,603	3.6 (3.4 to 3.9)
Actively on TNFi	137/24,055	5.7 (4.8 to 6.7)	476/55,654	8.6 (7.8 to 9.4)	394/118,801	3.3 (3.0 to 3.7)
Short-term exposure	74/11,671	6.3 (5.0 to 8.0)	316/35,204	9.0 (8.0 to 10.0)	216/66,404	3.3 (2.8 to 3.7)

^a^ACS, Acute coronary syndrome; IR, Incidence ratio; RA, Rheumatoid arthritis; TNFi, Tumour necrosis factor inhibitor. ‘Crude IR’ is the incidence per 1,000 person-years expressed as incidence rate (95% confidence interval). ^b^‘Ever exposed’ is all follow-up time after exposure was evaluated, ‘Actively on TNFi’ includes follow-up time until reported stop date for the TNFi plus 90 days and corresponding date for referents and ‘Short-term exposure’ is defined as actively on TNFi with follow-up limited to 2 years.

The fully adjusted Cox proportional hazards regression models resulted in significantly lower risk for ACS among the TNFi-exposed RA patients compared with the biologic-naïve RA referents, with an HR of 0.82 for the ever-exposed cohort (95% CI = 0.70 to 0.95) (Table 3). However, compared with the general population, the risk for ACS in both cohorts of RA patients was significantly increased: The HR (ever-exposed) for the TNFi-exposed patients was 1.61 (95% CI = 1.36 to 1.92), and it was 2.03 (95% CI = 1.80 to 2.29) for the biologic-naïve patients (Table 3). In regression models stratified by sex, age (<65 or ≥65 years), previous diagnosis of diseases carrying an increased risk for cardiovascular events (none or any) and time point of inclusion (2001 to 2005 or 2006 to 2010), significantly lower HRs were noted for TNFi-exposed compared to biologic-naïve RA patients in all subsets (Table 4). Further sensitivity analyses were performed, including patients with RA who started TNFi treatment on 1 January 2006 or later (*N* = 4,285, mean age 56.6 ± 12.7 years, female sex = 75.9%) and their matched biologic-naïve RA controls (*N* = 13,155), including data on medications started within 6 months prior to the matching date. Among the TNFi-exposed individuals, 93.3% (*N* = 4,090) had been treated with a DMARD and/or oral corticosteroid within 6 months before inclusion in the study, and, in the biologic-naïve cohort, the corresponding figure was 78.5% (*N* = 10,329). The baseline characteristics of the patients included in the sensitivity analysis are presented in Additional file 2. The results of these further adjustments are not markedly different from those of the main analysis, although the HRs in this smaller sample were statistically significant only for the risk window ‘actively on TNFi’ (Table 5).

**Table 3:** **Comparisons of risk for acute coronary syndrome between study cohorts**^**a**^

**Cohort/risk window**	**Biologic-naïve RA comparator**		**General population comparator**	
	**HR**^**b **^**(95% CI)**	**HR**^**c **^**(95% CI)**	**HR**^**b **^**(95% CI)**	**HR**^**c **^**(95% CI)**
TNFi-treated RA				
Ever-exposed	0.81 (0.69 to 0.94)	0.82 (0.70 to 0.95)	1.93 (1.66 to 2.25)	1.61 (1.36 to 1.92)
Actively on TNFi	0.71 (0.58 to 0.86)	0.73 (0.60 to 0.89)	1.73 (1.43 to 2.10)	1.50 (1.21 to 1.85)
Short-term exposure	0.75 (0.58 to 0.97)	0.78 (0.61 to 1.01)	2.07 (1.59 to 2.70)	1.65 (1.23 to 2.22)
Biologic-naïve RA comparator				
Ever-exposed	–	–	2.35 (2.11 to 2.63)	2.03 (1.80 to 2.29)
Actively on TNFi	–	–	2.41 (2.11 to 2.76)	2.10 (1.82 to 2.43)
Short-term exposure	–	–	2.63 (2.21 to 3.13)	2.27 (1.89 to 2.73)

^a^HR, Hazard ratio; RA, Rheumatoid arthritis; TNFi, Tumour necrosis factor inhibitor. ^b^Model adjusted for age, sex and county of residence. ^c^Model stratified for disability pension during the year before inclusion and adjusted for sex; age at time of inclusion; county of residence; year of inclusion; previous joint surgery; previous diagnosis of diabetes, hypertension, chronic obstructive pulmonary disease, cerebrovascular disease or other atherosclerotic disease; previous hospitalization for infectious disease; RA disease duration longer than 10 years (biologic-naïve and TNFi comparison); total number of days in hospital during 10 years prior to inclusion; sick leave; and education level.

**Table 4:** **Relative risk of acute coronary syndrome associated with tumour necrosis factor inhibitor**^**a**^

**Strata**	**Ever-exposed**			**Actively on TNFi**		
	**TNFi-exposed RA (ACS events/person-years)**	**Biologic-naïve RA (ACS events/person-years)**	**TNFi-exposed vs. biologic-naïve**^**b **^**(HR (95% CI))**	**TNFi-exposed RA (ACS events/person-years)**	**Biologic-naïve RA (ACS events/person-years)**	**TNFi-exposed vs. biologic-naïve**^**b **^**(HR (95% CI))**
Female	143/24,978	420/57,583	0.85 (0.70 to 1.03)	79/18,170	282/42188	0.70 (0.54 to 0.90)
Male	78/7,643	260/17,682	0.74 (0.57 to 0.96)	58/5,885	194/13466	0.72 (0.53 to 0.96)
Age <65 yr	111/25,208	331/55,041	0.76 (0.61 to 0.94)	74/18,969	250/42089	0.68 (0.52 to 0.89)
Age ≥65 yr	110/7,413	349/20,227	0.87 (0.70 to 1.07)	63/5,086	226/13,565	0.75 (0.57 to 0.99)
Cardiovascular risk factor present	70/5,520	259/15,321	0.78 (0.60 to 1.02)	43/3,898	185/11,154	0.67 (0.48 to 0.93)
No previous cardiovascular risk factor	151/27,101	421/59,946	0.86 (0.71 to 1.04)	94/20,157	291/44,500	0.77 (0.61 to 0.97)
TNFi start 2001 to 2005	175/22,505	517/48,870	0.81 (0.68 to 0.97)	106/15,627	344/33,726	0.72 (0.58 to 0.90)
TNFi start 2006 to 2010	46/10,116	163/26,398	0.77 (0.55 to 1.07)	31/8,427	132/21,928	0.64 (0.43 to 0.95)

^a^ACS, Acute coronary syndrome; RA, Rheumatoid arthritis; TNF, Tumour necrosis factor. ^b^The analyses were adjusted for age, sex and county of residence. The analysis stratified for sex was adjusted for age and county of residence. ‘Cardiovascular risk factor’ refers to any previous diagnosis of diabetes, hypertension and cerebrovascular and/or other atherosclerotic disease.

**Table 5:** **Sensitivity analyses adjusted for pharmacological treatment in rheumatoid arthritis patients included 1 January 2006 and later**^**a**^

**Risk window**	**TNFi-exposed RA patients ACS/person-years (*N*** **= 4,385)**	**Biologic-naïve RA patients ACS/person-years (*N*** **= 13,155)**	**General population comparator ACS/person-years (*N*** **= 21,925)**	**TNFi-exposed vs. biologic-naïve RA patients**		**TNFi-exposed RA patients vs. general population comparator HR**^**b **^**(95% CI)**	**Biologic-naïve RA patients vs. general population comparator HR**^**b **^**(95% CI)**
				**HR**^**b **^**(95% CI)**	**HR**^**c **^**(95% CI)**		
Ever exposed to TNFi	46/10,116	163/26,398	162/50,692	0.77 (0.55 to 1.07)	0.72 (0.51 to 1.02)	1.45 (1.05 to 2.02)	1.83 (1.47 to 2.27)
Actively on TNFi	31/8,427	132/21,928	128/41,757	0.64 (0.43 to 0.95)	0.64 (0.43 to 0.96)	1.21 (0.82 to 1.79)	1.85 (1.45 to 2.36)
Short-term exposure	22/5,986	107/18,229	102/33,574	0.69 (0.44 to 1.09)	0.64 (0.40 to 1.03)	1.27 (0.80 to 2.02)	1.83 (1.39 to 2.40)

^a^ACS, Acute coronary syndrome; HR, Hazard ratio; RA, Rheumatoid arthritis; TNFi, Tumour necrosis factor inhibitor. ^b^Adjusted for age, sex and county of residence. ^c^Adjusted for sex; age; county of residence; year of inclusion; diabetes mellitus (treatment and/or previous diagnosis); hypertension (treatment and/or previous diagnosis); previous diagnosis of chronic obstructive pulmonary disease, cerebrovascular disease or other atherosclerotic disease; previous hospitalization for infection; total number of days in hospital during 10 years prior to inclusion; RA disease duration more than 10 years; sick leave; disability pension; previous joint surgery; education level; and dispensed pharmacological treatment within 6 months prior to inclusion (acetylsalicylic acid, corticosteroids, disease-modifying antirheumatic drugs, lipid-lowering drugs, nonsteroidal anti-inflammatory drugs and coxibs). The frequency of the covariates in the RA cohorts is reported in Additional file 2.

## Discussion

In our nationwide, population-based cohort study, we observed a lower risk of incident ACS in patients with RA starting a first TNFi compared to patients with RA treated otherwise. However, the risk for a first-time ACS event among patients with RA, whether treated with TNFi or not, was 1.5 to 2 times higher than the risk in the general population. The association between TNFi treatment and risk of ACS was the most pronounced for patients being treated with ongoing TNFi therapy, but the point estimates were in the same order of magnitude in all of the subpopulations assessed, whether stratified by sex, age, previous occurrence of diagnoses reflecting cardiovascular risk factors or year of TNFi therapy start.

In several studies during recent years, researchers have evaluated the risk for ischaemic heart disease, myocardial infarction or ACS after TNFi therapy, with inconsistent results [12,14,15,17-21]. In our previous publication with our colleagues in the Swedish ARTIS Study Group [15], no statistically significant difference in risk for ACS was seen in early RA, without regard to TNFi exposure, but we did find a point estimate (HR = 0.80, 95% CI = 0.52 to 1.24) in keeping with that calculated in our present study. Two earlier observational studies have shown a decreased risk of myocardial infarction in RA patients after TNFi treatment compared to patients treated otherwise, with relative risks of 0.24 (95% CI = 0.06 to 0.95) [14] and 0.42 (95% CI = 0.21 to 0.81) [16]. Researchers in another study, with the outcome of ischaemic heart disease, reported a lower risk among TNFi-exposed patients (relative risk = 0.18 (95% CI = 0.05 to 0.61)) [12]. In a meta-analysis published in 2011 that included two of the studies cited above [13,16], two earlier Swedish studies [27,28] and abstract data from another two studies [29,30], the authors presented a pooled relative risk of 0.81 (95% CI = 0.68 to 0.96) for myocardial infarction associated with TNFi treatment [31]. To the best of our knowledge, no studies so far have shown any increase in the risk of coronary events after TNFi exposure. Despite the fact that we included patients from recent years, since 2001, after the introduction of TNFi and during a time span with remission or low disease activity as a goal for RA therapy, the risk for ACS among the RA patients compared to that in the general population was markedly increased. This indicates a remaining gap in risk for ischaemic heart disease between patients with RA and the general population during the calendar period in our study.

The strengths of this study include its large and nationwide setting and its comparatively large number of subjects and events. The high coverage of the Swedish Biologics Register [25] assured a low risk of misclassification of TNFi exposure. Furthermore, the register linkage allowed for adjustments regarding several possible confounders: cardiovascular risk factors, such as diagnosed hypertension, diabetes mellitus and prevalent atherosclerotic disease, as well as socioeconomic predictors of cardiovascular risk, such as education level, sick leave and disability pension. Information on prescribed medication was available in a subset of patients, allowing further adjustments for lipid-lowering, antidiabetic and antihypertensive therapy, DMARD therapy and treatment with drugs that might increase the risk of ACS (for example, corticosteroids, NSAIDs and coxibs). Access to data on general population referents provided us with the ability to contextualize the findings within the RA cohorts.

The study also has a number of limitations that should be acknowledged, however. We used the Swedish Patient Register to identify patients with RA. For the majority of individuals, therefore, specific RA-related data were not available. The frequency of joint surgery, DMARD and corticosteroid use (see Additional file 2), long RA disease duration and work-related disability in the RA cohorts (Table 1) indicate that the patients in the TNFi-exposed RA cohort were likely to have more severe or insufficiently controlled RA disease. It is unclear whether these adjustments were fully sufficient to adjust for disease activity, and therefore there might be remaining confounders (and confounding by indication, that is, that the start of TNFi treatment is *per se* an indicator of more severe RA disease). If RA activity were associated with the risk of ACS (and if other risk factors were comparable between the cohorts), a higher baseline risk level would be anticipated among the TNFi-exposed individuals. If so, this would have attenuated rather than inflated our findings. Regarding traditional cardiovascular risk factors, we had information on diagnoses of hypertension and diabetes mellitus and, in the sensitivity analyses, treatment for these risk factors and for hyperlipidaemia, but we had no information regarding smoking habits, blood pressure, glucose levels or anthropometric measurements. The frequency of risk factors (diagnosis of or treatment for) between the cohorts was similar, however.

## Conclusions

We found that patients with RA started on TNFi therapy in clinical practice had a lower risk of risk of ACS compared to RA patients treated otherwise. Compared with the general population, however, the risk for ACS in RA patients, regardless of TNFi treatment, was still increased. Further measures are thus needed to prevent premature coronary events in patients with RA, most likely by including disease control as well as other risk factor interventions.

## Abbreviations

ACS: Acute coronary syndrome; CVD: Cardiovascular disease; DMARD: Disease-modifying antirheumatic drug; HR: Hazard ratio; ICD: International Classification of Diseases; NSAID: Nonsteroidal anti-inflammatory drug; RA: Rheumatoid arthritis; TNFi: Tumour necrosis factor inhibitor.

## Competing interests

LL has received speaking fees from AbbVie and Bristol-Myers Squibb. JA has participated in an unrelated advisory board organised by Pfizer. SRD has no competing interests to declare. LJ has received consulting fees from AbbVie, Pfizer and UCB. The ARTIS Study Group conducts scientific analyses using data from the Swedish Biologics Register, which is run by the Swedish Society for Rheumatology. For the maintenance of this register, the Swedish Society for Rheumatology has received funding, independent of the conduct of these scientific analyses, from Merck, Bristol-Myers Squibb, Wyeth, AbbVie, UCB, Swedish Orphan Biovitrum (Sobi) and Roche. These companies had no influence on the study design, statistical analysis plan, data acquisition, analysis, interpretation of the results or the content of the manuscript. All final decisions resided with the investigators.

## Authors’ contributions

LL conducted data analysis and drafted the manuscript. JA contributed to the acquisition of the study data. JA, SRD and LJ contributed to the conception of the study. LL, JA, SRD and LJ participated in the design of the study, the interpretation of data and critical revision of the manuscript. All authors read and approved the final version of the manuscript.

## Authors’ information

The ARTIS (Anti-Rheumatic Therapy in Sweden) Study Group conducts scientific analyses using data from the Swedish Biologics Register. It also safeguards the quality and handling of the nationwide data collected. The following are the members of the ARTIS Study Group: Johan Askling, Lars Klareskog, Staffan Lindblad and Ronald von Vollenhoven (Karolinska Institute, Stockholm, Sweden); Eva Baecklund (Uppsala University, Uppsala, Sweden); Lars Cöster (Linköping University, Linköping, Sweden); Helena Forsblad and Lennart Jacobsson (Sahlgrenska Academy, Gothenburg, Sweden); Nils Feltelius (Chairman of the Medical Products Agency, Sweden); Pierre Geborek and Lars-Erik Kristensen (Lund University, Malmö and Lund, Sweden); and Solbritt Rantapää-Dahlqvist (Umeå University, Umeå, Sweden).

## Supplementary Material

Additional file 1 List of International Classification of Diseases (ICD) codes used for identification of the diagnoses covered in the study. Click here for file

Additional file 2 Baseline characteristics of TNFi-exposed patients who started TNFi on 1 January 2006 or later and their matched biologic-naïve comparators. Click here for file
