# Supplementary material for: The risk of acute coronary syndrome in rheumatoid arthritis in relation to tumour necrosis factor inhibitors and the risk in the general population: a national cohort study
Source: Arthritis Res Ther. 2014 Jun 18;16(3):R127. doi: 10.1186/ar4584 (PMC4095691; doi:10.1186/ar4584)
Supplement: Additional file 1 — List of International Classification of Diseases (ICD) codes used for identification of the diagnoses covered in the study. [file ar4584-S1.docx]

## ICD codes

#### Rheumatoid arthritis

ICD 10: M05, M06.0, M06.2, M06.3, M06.8, M06.9, M12.3

ICD 9: 714A-C, 714W, 719D

ICD 8:712.10, 712.20, 712.38, 712.39

#### Ischemic or congestive heart disease

ICD-10: I20-25, I50

ICD-9: 410-414, 428

ICD-8: 410-414, 427.00, 427.10, 428.9; ICD-7: 420, 434.1, 434.2, 434.4

#### Acute coronary syndrome:

Myocardial infarction ICD10: I21

Unstable angina ICD10: I20.0

#### Chronic obstructive and interstitial pulmonary disease

ICD 10: J41-44, J84.1, J84.9

ICD 9: 491-492, 496, 515-516

ICD 8: 490, 491.01, 491.02, 491.04, 492, 517

#### Diabetes mellitus

ICD 10: E10, E11

ICD 9: 250

ICD 8: 250

#### Hypertension

ICD 10: I10-15

ICD 9: 401-405

ICD 8: 400-404

#### Infections

ICD 10: A00-B99, G00-G02, G04.2, G05-G07, H66-H67, H70, J00-J22, J32, J34.0, J36, J38.3, J39.0-J39.1, K10.2, L00-L08, M00-M01, M46.2-M46.5, M86, N10, N30.0

ICD 9: 001-139, 320-322, 382-383, 460-466, 475, 480-487, 526E-526F, 590, 680-686, 711A, 711E, 730, 790H

ICD 8: 000-136, 320, 322, 381-383, 460-466, 470-474, 480-486, 501, 526.4, 590, 680-686, 710, 720, 782.9

#### Cerebrovascular disease

ICD 10: I60-69

ICD 9: 430-434, 436-438

ICD 8: 430-434, 436-438

Other atherosclerotic disease

(including arterial occlusion and stenosis of cerebral and precerebral arteries, transitoric ischemic attack, cerebral atherosclerosis, late effects of cerebrovascular disease, atherosclerosis, aortic aneurysm, other aneurysm or dissection, peripheral arterial disease, arterial embolism or thrombosis, atherosclerosis with gangrene)

ICD 10: G45, I65-I66, I67.2, I69-I72, I73.1, I73.9, I74, K55.0, K55.1

ICD 9: 433-435, 437A,B, 438, 440-442, 443B,X, 444, 557A,B

ICD 8: 432, 435, 437, 440-442, 443.10, 443.9, 444, 445
