# Supplementary material for: The risk of acute coronary syndrome in rheumatoid arthritis in relation to tumour necrosis factor inhibitors and the risk in the general population: a national cohort study
Source: Arthritis Res Ther. 2014 Jun 18;16(3):R127. doi: 10.1186/ar4584 (PMC4095691; doi:10.1186/ar4584)
Supplement: Additional file 2 — Baseline characteristics of TNFi-exposed patients who started TNFi on 1 January 2006 or later and their matched biologic-naïve comparators. [file ar4584-S2.docx]

| **Baseline characteristics for patients included in sensitivity analysis** | | | | |
| --- | --- | --- | --- | --- |
| TNFi exposed patients who started TNFi 2006-01-01 or later and their matched biologics-naïve  comparators. Values represent number and percentage unless otherwise stated. | | | | |
|  | **TNFi exposed (n=4385)** | | **Biologics-naive (n=13155)** | |
|  | **n** | **%** | **n** | **%** |
| Inclusion age (mean of years, SD) | 56.6 | 12.7 | 56.8 | 12.8 |
| Female sex | 3326 | 75.9 | 9978 | 75.9 |
| **Prevalent diagnoses:** |  |  |  |  |
| Diabetes | 235 | 5.4 | 754 | 5.7 |
| Hypertension | 557 | 12.7 | 1723 | 13.1 |
| Chronic obstructive pulmonary disease | 111 | 2.5 | 399 | 3.0 |
| Cerebrovascular disease | 109 | 2.5 | 415 | 3.2 |
| Other cardiovascular disease | 314 | 7.2 | 1093 | 8.3 |
| Infection (hospitalisation for) | 960 | 21.9 | 3089 | 23.5 |
| Joint surgery | 989 | 22.6 | 2223 | 16.9 |
| **Socioeconomic factors:** |  |  |  |  |
| Disability pension | 1294 | 29.5 | 3523 | 26.8 |
| Sick leave, part time | 1119 | 25.5 | 2657 | 20.2 |
| Sick leave, full time | 101 | 2.3 | 222 | 1.7 |
| RA disease duration >10 years | 704 | 16.1 | 1582 | 12.0 |
| **Dispensed, prescribed drugs during 6 months prior to inclusions:** |  |  |  |  |
| Acetylsalicylic acid | 256 | 5.8 | 901 | 6.8 |
| Corticosteroid | 2473 | 56.4 | 4737 | 36.0 |
| Antidiabetic drug(s) | 252 | 5.7 | 726 | 5.5 |
| Antihypertensive drug(s) | 1326 | 30.2 | 3972 | 30.2 |
| Lipidlowering drug(s) | 351 | 8.0 | 1221 | 9.3 |
| NSAID | 2469 | 56.3 | 5734 | 43.6 |
| Coxib | 213 | 4.9 | 448 | 3.4 |
| DMARD | 3793 | 86.5 | 9406 | 71.5 |
| Methotrexate | 3248 | 74.1 | 7768 | 59.1 |
| Sulphasalazine | 1084 | 24.7 | 1884 | 14.3 |
| Leflunomide | 268 | 6.1 | 267 | 2.0 |
| Ciklosporin A | 107 | 2.4 | 121 | 0.9 |
| Azathioprin | 94 | 2.1 | 144 | 1.1 |
| Gold salt | 33 | 0.8 | 82 | 0.6 |
| Chloroquine | 549 | 12.5 | 1214 | 9.2 |
| Synthetic combination therapy | 1308 | 29.8 | 1807 | 13.7 |
| Corticosteroid and DMARD in combination | 2176 | 49.6 | 13155 | 29.0 |
| Corticosteroid or DMARD therapy | 4090 | 93.3 | 10329 | 78.5 |
| **Medication for and/or diagnosis^1^ listed of:** |  |  |  |  |
| Diabetes | 297 | 6.8 | 902 | 6.9 |
| Hypertension | 1370 | 31.2 | 4132 | 31.5 |

^1^Includes treatment and/or diagnosis of diabetes and hypertension, respectively. The figures differ from prevalent diagnosis and dispensed medication as diagnoses in primary care are not included in the Patient Register, all co-morbidity might not be listed as contributory diagnoses in specialist clinics and furthermore, patients might have a diagnosis without receiving any treatment.
